# Mismatch Repair Deficiency and Somatic Mutations in Human Sinonasal Tumors

**DOI:** 10.3390/cancers13236081

**Published:** 2021-12-02

**Authors:** Lena Hieggelke, Carina Heydt, Roberta Castiglione, Jan Rehker, Sabine Merkelbach-Bruse, Cristina Riobello, José Luis Llorente, Mario A. Hermsen, Reinhard Buettner

**Affiliations:** 1 Institute of Pathology, University Hospital Cologne, University of Cologne, 50937 Cologne, Germany; lena.hieggelke@uk-koeln.de (L.H.); carina.heydt@uk-koeln.de (C.H.); jan.rehker@uk-koeln.de (J.R.); sabine.merkelbach-bruse@uk-koeln.de (S.M.-B.); 2 Institute of Pathology, Klinikum Stuttgart, 70174 Stuttgart, Germany; r.castiglione@klinikum-stuttgart.de; 3 Department of Head and Neck Oncology, Instituto de Investigación Sanitaria del Principado de Asturias, 33011 Oviedo, Spain; cristinarisu15@gmail.com (C.R.); mhermsen@hca.es (M.A.H.); 4 Department of Otolaryngology, Hospital Universitario Central de Asturias, 33011 Oviedo, Spain; llorentejlx@gmail.com

**Keywords:** sinonasal squamous cell carcinoma, microsatellite instability, mismatch repair deficiency, EGFR

## Abstract

**Simple Summary:**

Sinonasal carcinomas are rare tumors with an overall poor prognosis. Due to limitations in local therapeutic approaches, systemic neo-adjuvant or adjuvant therapies are becoming increasingly important in order to improve patient outcome. This study aimed to examine potentially therapeutic targetable molecular alterations in different sinonasal tumors, including deficiency in mismatch repair proteins and microsatellite instability as well as driver mutations. According to our results, immunohistochemical (IHC) analysis of mismatch repair (MMR) proteins and sequencing-based panel analysis should be integrated into the diagnostics of clinically aggressive inverted sinonasal papilloma (ISP) and sinonasal squamous cell carcinoma (SNSCC) in order to enable the therapeutic possibility of a targeted therapy.

**Abstract:**

Due to limitations in local therapy approaches for sinonasal tumors, improvement in systemic therapies plays a pivotal role for prolongation of the patient’s survival. The aim of this study was to examine potential biomarkers, including deficiency in mismatch repair proteins (dMMR)/microsatellite instability (MSI-H) in sinonasal cancers and their precancerous lesions. A comprehensive analysis of 10 sinonasal cancer cell lines by whole exome sequencing, screening 174 sinonasal tumors by immunohistochemistry (IHC) for mismatch repair deficiency and next generation sequencing (NGS) of 136 tumor samples revealed a dMMR/MSI-H sinonasal squamous cell carcinoma (SNSCC) cell line based on a somatic missense mutation in *MLH1* and an overall frequency of dMMR/MSI-H SNSCC of 3.2% (4/125). Targetable *EGFR* mutations were found in 89.3% (25/28) of inverted sinonasal papilloma (ISP) and in 60% (6/10) of ISP-associated carcinomas. While *PIK3CA* and *EGFR* mutations were not mutually exclusive, *KRAS* mutated tumors were an *EGFR*-wildtype. The effect of potential driver mutations in *FGFR2, FGFR3, BRAF, HRAS, MAP2K1, PTEN, NOTCH1* and *CARD11* need further investigation. Our results suggest that biomarker testing, including MMR-IHC and NGS panel analysis, should be integrated into the diagnostics of clinically aggressive ISPs and SNSCC to assess prognosis and facilitate therapeutic decisions.

## 1. Introduction

Nasal cavity and paranasal sinus cancers are a group of rare cancers, representing up to 5% of all head and neck cancers with an annual incidence of approximately 1 case per 100,000 inhabitants worldwide and an average age between 50 and 60 years [1]. With regard to their poor prognosis, it is of great interest to improve our knowledge and therapeutic options of these cancers. Based on unspecific or mild symptoms at early stages of tumor development, sinonasal cancers have a prolonged diagnostic latency [2,3]. Over the past decade, advances in imaging techniques, surgical approaches (especially endoscopic interventions) and radiotherapy have contributed to better management of patients with sinonasal tumors [4,5]. Due to the anatomic area closely related to the central nervous system, local treatment options are limited and systemic neoadjuvant or adjuvant therapies have a pivotal role in improving the outcome of patients treated with a curative intent [6]. The management of recurrent and incurable sinonasal carcinomas is not yet standardized [7]. The implementation of targeted therapeutic options potentially leads to an improved overall disease-specific survival. With this intention, recent studies elucidated the histomorphological and genetic heterogeneity of sinonasal cancers. The most common histological types are sinonasal squamous cell carcinomas (SNSCC), representing slightly more than 50%, and adenocarcinomas, while the remaining tumors include a miscellany of neuroendocrine carcinomas, neuroectodermal neoplasms, salivary gland tumors, undifferentiated carcinomas and sarcomas, whereas the most common primary sites are the nasal cavity and maxillary sinus [8]. 

Poorly differentiated tumors are challenging, and ancillary immunohistochemical stainings are required to exclude the differential diagnosis of a lymphoma, a sarcoma and a mucosal melanoma and an olfactory neuroblastoma (ONB) for a small blue round cell carcinoma. Recently, the group of poorly and undifferentiated carcinomas were further investigated and molecular subgroups were defined. In these cases, differential diagnosis of a basaloid squamous cell carcinoma [9] and a lymphoepithelial carcinoma [10] should be considered, as well as a solid adenoid cystic carcinoma (ACC), which often carries an MYB or MYBL1 translocation [11]. An ACC without an MYB translocation should be distinguished from a newly described subtype of human papillomavirus-(HPV)-related carcinoma with adenoid cystic-like features (also known as Human Papillomavirus-Related Multiphenotypic Sinonasal Carcinoma), which is particularly associated with HPV type 33 [12,13]. The diagnosis of a sinonasal undifferentiated carcinoma (SNUC) became a diagnosis of exclusion and must also be distinguished from an aggressive, poorly differentiated carcinoma with round blue cells and an abrupt keratinization NUT (NUclear protein in Testis) carcinoma, which can be diagnosed by at least diffuse nuclear staining of > 50% of tumor cells for a NUT protein [14,15] and a SMARCB1 (INI-1)-deficient sinonasal carcinoma or sinonasal neuroendocrine carcinoma (SNEC) [16,17,18]. 

Even for SNSCCs with well to moderate differentiation processes that are driving tumorigenesis are complex, and the pathogenesis is not completely understood yet. Currently, two major oncogenic pathways were described for SNSCCs—infection by high-risk human papilloma virus (HR-HPV) and constitutively activating Epidermal-Growth-Factor-Receptor (*EGFR*) mutations. HR-HPV infection positive SNSCCs were reported in 7.5–25% of SNSCC [19,20,21]. However, the prognostic impact of HPV infections on a clinical outcome remains unclear [20,22,23]. SNSCCs, which harbor an *EGFR* mutation most frequently in exon 20, were described in the absence of an HR-HPV infection and predominantly arose from inverted sinonasal papillomas (ISPs), a locally aggressive subtype of sinonasal papillomas (SPs) [19,24]. This fact led to the consideration that ISPs are part of the same spectrum of tumor evolution and opened up a therapeutic option for this tumor entity [25,26]. In addition, recurrent somatic variants including *KRAS*, *TP53, CDKN2A, NFE2L2, PIK3CA, NOTCH1* and *PIK3R1* were already described in SNSCC [27], whereas *TP53, CDKN2A* and *KRAS* mutations were recurrent in intestinal-type adenocarcinoma (ITAC) [28]. Additionally, there is another group of systemically effective therapeutic agents and immune checkpoint inhibitors (ICIs) for therapeutics that specifically attack molecular structures that result from mutations in the tumors; this includes programed cell death protein 1 (PD-1) inhibitors, which have redefined the treatment paradigm of various types of advanced cancers, including patients with recurrent/metastatic HNSCC [29] in the last few years.

Microsatellite instability (MSI) is a form of global genomic hypermutation, leading to length alterations in short repetitive DNA sequences, as well as single nucleotide substitutions and frameshifts both in coding and non-coding genomic sequences. MSI is a consequence of genetic or epigenetic defects in genes encoding DNA mismatch repair (MMR) proteins, referred to as dMMR [30]. MSI secondary to germline mutations in DNA MMR proteins is the molecular fingerprint of Lynch syndrome, a dominantly inherited cancer syndrome, while epigenetic inactivation of these genes is more commonly found in sporadic MSI tumors [30], including colorectal, endometrial, gastric, small intestine, urothelial, central nervous system and sebaceous gland neoplasms [31]. 

The MSI/MMR status of cancer is now considered as an important predictor for sensitivity to ICI treatment [32,33,34], underlined by the Food and Drug Administration (FDA)’s first approval of immune checkpoint inhibitors for refractory, adult and pediatric tumors, based on a common biomarker (dMMR or MSI high (MSI-H)) rather than the primary site of origin [35]. 

For systematic analysis of the genomic background in sinonasal carcinoma, we analyzed 10 cell lines of sinonasal cancers by whole exome sequencing. In order to extend our findings, we analyzed 141 samples of sinonasal tumors, including SPs by NGS-based targeted gene analysis and by ancillary tests of dMMR, MSI, p16 and HPV, with the ambition to elucidate molecular tumor subgroups for prognosis prediction and an individualized therapeutic strategy for patients with sinonasal cancer.

## 2. Materials and Methods

### 2.1. Tumor Specimens 

The study cohort of human sinonasal tumors included 220 tumor samples (141 from University Hospital of Cologne/Germany, 79 from University Hospital of Oviedo/Spain). Histological tumor subtype, material type and origin as well as analysis performed are summarized collectively with personal attributes (sex, age at diagnosis, tumor location) in Table S1.

The cohort of 141 formalin-fixed and paraffin embedded (FFPE) tumor samples included 43 keratinizing sinonasal squamous cell carcinoma (SNSCC), 10 non-keratinizing SNSCC, 10 inverted sinonasal papilloma (ISP)-associated SNSCC, 6 sinonasal undifferentiated carcinoma (SNUC), 7 intestinal-type adenocarcinoma (ITAC), 11 adenocarcinoma with neither intestinal nor salivary gland differentiation (SNAC), 11 adenoid cystic carcinoma (ACC), 2 sinonasal neuroendocrine carcinoma (SNEC), as well as 28 inverted sinonasal papillomas (ISP), 6 exophytic sinonasal papillomas (ESP) and 1 oncocytic sinonasal papilloma (OSP), diagnosed from January 1990 to March 2021. Tissue samples were obtained as part of routine clinical care under approved ethical protocols compiled with the Ethics Committee of the Medical Faculty of the University of Cologne (Ethics-No. 13-091, BioMaSOTA) and by the Institutional Ethics Committee of the Hospital Universitario Central de Asturias and the Regional CEIC from Principado de Asturias (approval numbers: 83/17 for project PI17/00763 and 07/16 for project CICPF16008HERM), and informed consent was obtained from each patient. Personal attributes (sex, age at diagnosis, tumor location) were collected from institutes internal databases, and all samples were anonymized. 

We obtained scans of tissue micro arrays (TMAs) (3 cores from each specimen) of 43 SNSCC and archived the corresponding formalin-fixed paraffin-embedded material (FFPE) of 26 SNSCC cases and additional FFPE samples of 4 SNUC and 6 SNEC. 

The presence of HPV was assessed for 141 tumors via HPV-specific PCR and immunohistochemistry of the marker p16INK4A (Clone: JC2, Zytomed, Berlin, Germany) in 161 tumors, as reported previously [36]. For the HPV-specific PCR, DNA was analyzed with the HPV direct 3.5 Kit (Chipron, Berlin, Germany) following the manufacturer’s instructions. Only patients with a positive PCR result were categorized as HPV-positive.

### 2.2. Sinonasal Cancer Cell Lines 

Immortal sinonasal squamous cell cancer cell lines (SCCNC) SCCNC1, SCCNC5, SCCNC6 and SCCNC7 were set up from a primary keratinizing SNSCC, while SCCNC6-Rec and SCCNC6-LN were derived from recurrence and lymph node metastasis, respectively, of the same patient as SCCNC6. SCCNC8 and SCCNC9 concerned primary keratinizing SNSCC cultures. Immortal cell lines SCCNC4 and ITAC3 were established from a primary ISP-associated SNSCC [29] and a primary colonic-type ITAC, respectively [37]. In the following, SCCNC cell lines were abbreviated as NC.

### 2.3. Immunohistochemistry of Mismatch Repair (MMR) Proteins 

We performed an immunohistochemical analysis on multitumor TMAs and whole slides. All tumors were stained for MLH1, MSH2, MSH6, PMS2 (using MLH1 (Clone: M1, Host: mouse, Ventana Roche, Tucson, Arizona), MSH6 (Clone SP93, Ventana Roche), PMS2 (Clone: A16-4, Ventana Roche) and MSH2 (Clone: G219–1129, Ventana Roche)) on Ventana Benchmark stainers. 3,3′-Diaminobenzidine (DAB) was used as a chromogen and hematoxylin as a counterstain. Staining results were assessed independently by two observers (LH, RB). Tumors with clearly strong nuclear positive tumor cells compared to the internal positive control (immune cells) were classified as pMMR. Tumors that showed significantly reduced nuclear staining compared to intratumoral immune cells or tumors that displayed complete loss of an MMR protein were classified as dMMR.

### 2.4. Targeted Panel NGS

The tumor area (tumor cell content >10%) of each sample was highlighted on a hematoxylin and eosin (H&E) stained slide. DNA was extracted from the tumor area of unstained 10-μm thick slides by manual micro-dissection using the Maxwell 16 FFPE Plus Tissue LEV DNA Purification Kit (Promega, Madison, WI, USA) on the Maxwell 16 (Promega) following the manufacturer’s instructions. The quality and quantity of isolated DNA was assessed with a quantitative real-time PCR (qPCR) kit (GoTaq qPCR Master Mix; Promega). Isolated DNA was amplified with customized GeneRead DNAseq Targeted Panels V2 (Qiagen, Hilden, Germany) and the GeneRead DNAseq Panel PCR Kit V2 (Qiagen) following the manufacturer’s instructions. The three GeneRead DNAseq Targeted Panels V2 used contained subsets of the following genes: *ATK1*, *ALK*, *BRAF*, *CARD11*, *CDK4*, *CDKN2A*, *CTNNB1*, *C15orf23*, *DDR2*, *EGFR*, *ERBB2*, *FGFR1*, *FGFR2*, *FGFR3*, *GNA11*, *GNAQ*, *HRAS*, *IDH1*, *IDH2*, *KEAP1*, *KIT*, *KNSTRN*, *KRAS*, *MAP2K1*, *MET*, *NFE2L2*, *NOTCH1*, *NRAS*, *OXA1L*, *PDGFRA*, *PIK3CA*, *PTEN*, *SMAD4*, *RAC1*, *STAT3* and *TP53* (Table S2). After end-repair and adenylation, NEXTflex DNA Barcodes (HISS Diagnostics, Bochum, Germany) were ligated. Barcoded libraries were amplified, and final library products were quantified, diluted and pooled in equal amounts. In total, 12 pM of the constructed libraries were sequenced on the MiSeq (Illumina, San Diego, CA, USA) with a MiSeq reagent kit V2 (300-cycles) (Illumina) following the manufacturer’s recommendations. Data were exported as FASTQ files. Alignment and annotation were done using a modified version of a previously described method [10]. A 5% cut-off for variant calls was used, and results were only interpreted if the coverage was >200× (Table S3).

### 2.5. Microsatellite Instability (MSI) Analysis

Microsatellite instability was analyzed by a reference panel marker test (BAT25, BAT26, D5S346, D2S123, D17S250) as previously described [38]. Tumors showing no instabilities in these markers were classified as microsatellite-stable (MSS). Tumors having two or more instable markers were classified as MSI-high (MSI-H).

### 2.6. Whole Exome Sequencing of Sinonasal Cancer Cell Lines 

Cell lines were cultured in 75 cm^2^ cell culture flasks (Corning, Corning, NY, USA) with a three-times a week changed medium (DMEM.F12, Thermo Fisher Scientific, Waltham, MA, USA) under stable conditions (37 °C and 5% CO^2^) in the incubator. Cell pellets were prepared and digested overnight by incubation with proteinase K (Merck KGaA, Darmstadt, Germany). DNA isolation was performed with the Maxwell 16 LEV Blood DNA Purification Kit (Promega) on the Maxwell 16 (Promega) following the manufacturer’s instructions. Extracted DNA was quantified using the Qubit dsDNA HS Assay (Thermo Fisher Scientific) on the Qubit 2.0 Fluorometer (Thermo Fisher Scientific, Waltham, MA, USA) and prepared for shearing according to the SureSelect XT Target Enrichment System Manual (Agilent, Santa Clara, CA, USA). In total, 200 ng of DNA was sheared on the Covaris E220 Focused-Ultrasonicator (Woburn, MA, USA) to a fragment size of 150 bp using the 8 microTUBE–50 Strip AFA Fiber V2 following the manufacturer’s instructions. The treatment settings were the following: peak incident power (W): 175; duty factor: 10%; cycles per burst: 200; treatment time (s): 200; temperature (°C): 7; water level: 6. For library preparation, the SureSelect XT Reagent Kit (Agilent, Santa Clara, CA, USA) was used following the manufacturer’s instructions. In brief, pre-enriched adapter-ligated libraries were prepared. Subsequently, Human all Exon v6 capture probes were hybridized to target sequences to allow for sequence enrichment using streptavidin beads. Post-enriched libraries were quantified, pooled and sequenced on a NextSeq 500 (Illumina San Diego, CA, USA). Quality of the NextSeq 500 (Illumina) sequencing runs were assessed with the Illumina Sequencing Analysis Viewer (Illumina). FASTQ files were generated using bcl2fastq Conversion Software (Illumina).

A tumor mutation burden (TMB) analysis was performed according to omitting molecular consensus read calling and realignment, as the Agilent SureSelect XT kit does not allow for Unique Molecular Identifier (UMI)-based deduplication [39].

We determined microsatellite instabilities with MSIsensor-pro running in tumor-only mode with a panel of normals [40].

The resulting alignment files were subsequently used for the identification of driver variants, detection of MSI and CNV analysis. CNVs were called according to the GATK ‘best practices workflow’: ‘Somatic copy number variant discovery’. Alignment files of both tumor and matching normal were analyzed. Only CNVs that could not be found in the matching normal were reported in the manuscript. Copy number loss was defined as 2 log copy ratio change −1 to 0. 

Mutect2 was used in the tumor-normal mode, followed by the GATK FilterMutectCalls tool. The resulting vcf files were annotated with ANNOVAR [41]. The annotated files were filtered with bcftools [42] for variants that were marked with ‘PASS’ by GATK. We achieved at 100-fold a mean sequence coverage of 24,054,454.95 bases in coding exons (range 12,965,569–30,533,500), which corresponds to a mean average coverage of 70.25% and had a minimum sequencing depth of 10 reads in the tumor and 8 reads in the normal. 

A mutational profile analysis by a web tool-based approach, Mutational Signatures in Cancer (MuSiCa) [43], was performed. 

In total, 3564 variants affecting 2893 different genes with a mean allelic depth (AD) of 143.4 were found. These genes were filtered according to the following criteria: (1) An allelic fraction (AF) of greater then 0.05, (2) listed in the Cancer Gene Census (CGC) of Catalogue Of Somatic Mutations In Cancer (COSMIC) and (3) genes coding for proteins participate in DNA repair. If criterion 1 was considered, the number of genes was reduced to 2820. Recurrent mutated genes were defined as genes mutated in more than one cell line from different individuals. For assessment of variants, the following databases were used: ClinVar database: http://www.ncbi.nlm.nih.gov/clinvar/ accessed date 1 September 2021, COSMIC: https://cancer.sanger.ac.uk/cosmic/ accessed date 1 September 2021.

### 2.7. Statistical Analysis and Visualization 

Qualitative variables were summarized by count (N) and percentage and quantitative variables by mean and standard deviation (SD). Bivariable association was evaluated by a Pearson correlation and chi-square test for trend. Clustered heatmaps of mutational signature were created using R version 4.1.1 (2021-08-10). Plots were created using a web-based tool of cBioPortal [44,45], IBM SPSS Statistics 27 (2017, IBM Armonk, North Castle, NY, USA) and Graphpad Prism 9.2.0 (2021, GraphPad Software, La Jolla, CA, USA) for Windows.

## 3. Results

### 3.1. Genomic Profiling of Cell Lines Revealed a MMR Deficient SNSCC 

In order to identify alterations in the cancer genome of the most frequent sinonasal tumor types, SNSCC and ITAC, we performed whole exome sequencing (WES) of 10 patient-derived tumor cell lines, including seven primary SNSCC cell lines (NC1, NC4, NC5, NC6, NC7, NC8, NC9) as well as one matched cell line of lymph node metastasis (NC-6LM) and relapsed tumor (NC6-relapse) and one ITAC cell line (ITAC3).

**Figure 1:**
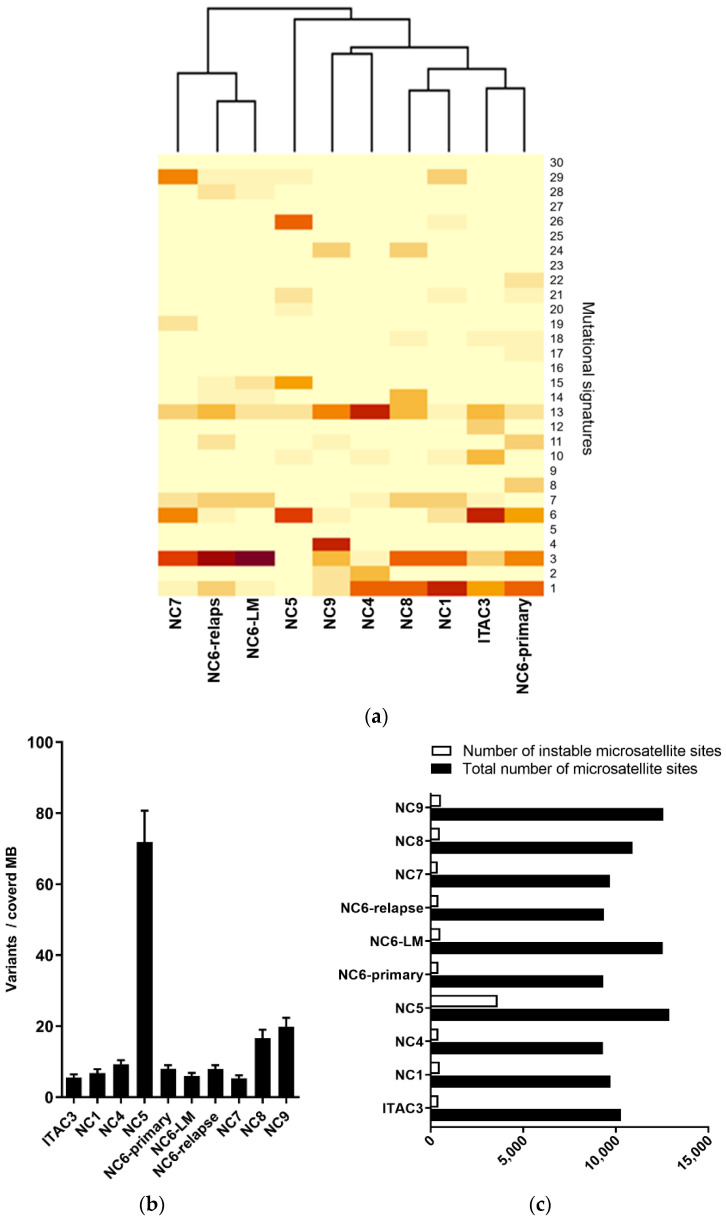
Mismatch repair deficiency signature in SNSCC cell line. (**a**) Mutational signature analysis based on WES of 6 patient-derived sinonasal squamous cell carcinoma (SNSCC) cell lines (NC) and one patient’s lymph node metastasis (NC6-LM)/tumor recurrence (NC6-relapse). A sinonasal intestinal type adenocarcinoma (ITAC3) revealed a mismatch repair-deficient signature of NC5 (signature 6, 15, 20, 26). NC6-primary, ITAC3, NC1, NC4 and NC8 clustered with the highest impact of age-associated signature (1). NC6-primary, ITAC3, NC1 and NC8 showed alteration resembling homologous recombination (HR) repair system deficient tumors (signature 3), which was the predominant signature of NC6-relapse, NC6-LM and NC7 (signature 3). NC9 mostly collected smoking-associated mutations (signature 4). (**b**) Tumor mutational burden (TMB) analysis of cell line samples revealed an average of variants per covered mega base (MB) of 13.67 for non-synonymous and for all coding sequencings of 17.784, confirming a TMB-high status for NC5. (**c**) Analysis of microsatellite sites in WES data of cell lines elucidated an abnormal percentage of instable microsatellite regions compared to all analyzed sites in individual normal tissue control, classifying the NC5 as a highly microsatellite instable (MSI-H) carcinoma.

A mutational profile revealed a predominance of nucleotide transitions over transversions with preferential targeting of C and T, which was particularly found in ITAC3, NC1, NC5, NC6-primary, NC6-LM and NC6-relapse. The mutational signatures were grouped according to COSMIC Signatures (Figure 1a). Here, NC6-primary, ITAC3, NC1, NC4 and NC8 clustered with the highest impact of age-associated signature 1. NC6-relapse, NC6-LM and NC7 displayed a clustering based on signature 3, which was also found in NC6-primary, ITAC3, NC1 and NC8. Signature 3 is defined by an elevated number of large (longer than 3bp) insertions and deletions with overlapping microhomology at breakpoint junctions and are known to be associated with mutations in *BRCA1* and *BRCA2* or deficiency of a homologous recombination (HR) repair system in breast, pancreas and ovarian cancer [46,47]. NC6-relapse and NC6-LM cell lines derived from an NC6-primary tumor patient 21 months after resection and radiotherapy. For radiation-associated secondary malignancies, a significant excess of long-segment-deletions relative to insertions was already described, which can typically be found in *BRCA1* or *BRCA2* germline-deficient breast tumors [48]. The fact that NC6-LM and -relapse clustered based on signature 3 might indicate spreading of a cancer clone persisting during radiation therapy. Only the NC9 cell line exhibited a mutational signature of tobacco smoking-related genetic defects, signature 4, which matched patient’s history of smoking. A clear pattern of signatures 6, 15, 20 and 26 was detected in NC5. This signature profile is associated with high numbers of small (shorter than 3bp) insertions and deletions at mono/polynucleotide repeats, which indicated a defect in the DNA mismatch repair system of NC5 [49]. 

By analyzing the TMB of the cancer cell lines, we detected a mean number of non-synonymous mutations per covered MB of 13.7 and an overall variation average of 17.8. TMB of the NC6-LM (6.1) and NC6-relapse (7.1) cell lines were not increased compared to NC6-primary (8.0). Thereby, the NC5 cell line revealed by far the highest TMB with 63.1 non-synonymous mutations per covered MB (total 80.7 mutations/MB), which can indicate a high-level microsatellite instability (MSI-H) (Figure 1b). 

In order to prove this hypothesis, we analyzed microsatellite regions in WES data of the tumor cell lines compared to their individual normal tissue/blood controls. Given a range in a panel of normal tissue analyzed previously, we determined a cut-off value for MSI of 7%. In a total number of suspicious sites (mean 9995.5; from 9299 to 12,902), we detected an average rate of instable microsatellite regions of 4.3%. The percentage of MSI sites in 9 of 10 tumor cell lines was in a limited range from 3.9% to 4.3%, and only NC5 showed a rate of 28%, which confirmed NC5 as an MSI-H carcinoma (Figure 1c).

### 3.2. Mismatch Repair Deficiency Based on Somatic MHL1 Mutation 

**Figure 2:**
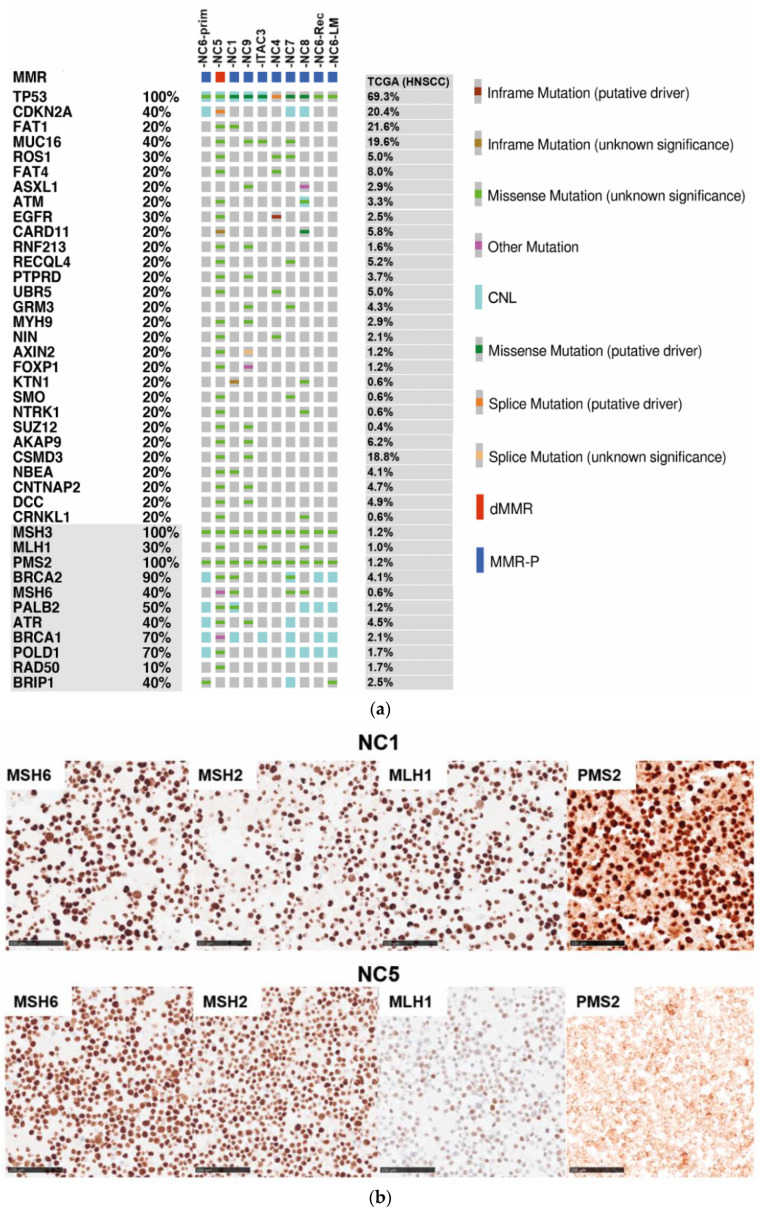
Putative driver mutations in cancer-associated genes and genes of DNA repair of sinonasal cell lines. (**a**) Recurrently mutated cancer genes in the cell lines were listed in comparison to TCGA data of head and neck squamous cell cancer (HNSCC); genes belonging to the DNA repair system shaded in gray. Potential driver mutations were found in the *TP53*, *EGFR*, *CDKN2A*, *CARD11* and *KTN1* genes. Copy number loss (CNL) of *BRCA2* and genes belonging to the homologous DNA repair (HR) as well as *TP53* and *CDKN2A* were found in the cancer cell lines. Mismatch repair genes frequently carried gene variations, which were classified as benign according to ClinVAR database, except for the NC5′s *MLH1* mutation, a somatic mutation with an allelic fraction of 99.8%, situated in the coding sequence for the ATPase region of MLH1 protein. (**b**) Immunohistochemical staining of MMR proteins revealed significant loss of MLH1 expression compared to the other cell lines (regular expression in NC1), followed by a complete loss of PMS2 protein expression but regular expression of MSH6 and MSH2. Scar bar 100 µm.

Considering variants in tumor cell lines with a mean allelic depth (AD) of 143.4, we found 3564 variants affecting 2893 different genes. A total of 3448 mutations had an allelic fraction (AF) greater than 0.05% (mean 344.8, SD 588.98). In line with our previous findings, exonic frameshift insertions occurred most frequently in NC5 (101/121, 83.5%, mean of all cell lines 13.5, SD 31.1), whereas the overall predominant mutation type was a non-synonymous single nucleotide variation (SNV): missense mutation (mean: 298.5, SD 486.8) and non-frameshift substitution (mean 4.29, SD 1.7). SNVs with the consequence of a premature stop codon (nonsense mutation) were detected in all cell lines (mean 16.4, SD 18.9), but NC5 (67/164, 40.9%) and NC9 (34/164, 20.7%) harbored the most. In addition, we found splice site mutations in all cell lines (mean 14.7, SD 23.9), with a ratio of splice site mutation to exonic mutation of 1:22.5. Based on the mutation profile, the NC6-LM and NC6-relapse cell line showed overlaps to NC6-primary (Pearson r 0.08) and a positive correlation to each other (Pearson r 0.69, *p* < 0.001). Thus, we concluded that NC6-LM and NC6-relapse were clonally related to each other, arising from a cancer clone of NC6-primary, which persisted after radiation. Based on filtering criteria, recurrent genetic aberrations were found in 235 genes. Twenty-nine known cancer genes were affected and listed compared to mutational frequency data of head and neck squamous cell cancer (HNSCC) from The Cancer Genome Atlas (TCGA) (Figure 2A). According to the mutational signatures, which suggest a deficiency in the DNA repair system, DNA mismatch and homologous recombination repair (HR) genes were analyzed for alterations. Copy number variations (CNV) of tumor samples normalized to a panel of normals and their individual normal tissue control were examined for the genes of interest. As expected, NC5 showed most mutations in the filtered genes (28/31). In total, there were 76 mutations in the 31 mutated genes. Most of them were missense mutations (59/76), followed by splice site variants (7/76), nonsense mutations (7/76), frameshift insertion (2/80) as well as one frameshift deletion. All of the cancer cell lines harbored a *TP53* mutation, including five known inactivating missense mutations, four missense mutations of unknown significance and one likely pathogenic splice site variation (NM_000546.5: c.559+1G>T). The NC6-primary, NC6-LM and NC6-relapse contained the same *TP53* mutation (NM_000546:p.P151S) and no additional mutation in the filtered cancer genes. NC4, as an SNSCC derived from a patient with inverted papilloma (ISP) in medical history, showed a typical *EGFR* mutation in exon 20 (NM_005228.5: c.2303_2311dup, p.S768_D770dup), whereas the *EGFR* mutation of NC5 was in exon 6. 

Another putative driver mutation was detected in *CARD11* of NC8 (AF 56.8%) situated in the coiled-coil domain of the CARD11 protein. Additionally, a *CARD11* in-frame mutation was found in NC5. The oncogenic potential, based on constitutive activation of the N nuclear factor kappa-light-chain-enhancer of activated B cells (NfķB) pathway, has already been described in diffuse large B-cell lymphoma [50], and an increased gene expression in HNSCC was shown previously [51]. 

For HR-genes, copy number losses (CNL) on chromosome (chr) 17 comprising *BRCA1* in NC1, NC6-primary, NC6-relapse, NC6-LM, NC7 and ITAC3 as well as on chr13, including *BRCA2* in NC6-primary, NC6-relapse, NC6-LM were detected. In addition, DNA segment deletions, including *ATR* in NC7 and NC6-primary and *ATM* in NC8, appeared. Further CNL of the gene regions comprising *PALB2* and *POLD1* were found in NC6-primary, NC6-relapse and NC6-LM. Heterozygous deletions of *TP53* (4/10), *CDKN2A* (3/10) and *ATM* (1/10) were found. The mutational analysis of MMR and HR-genes revealed 60 exonic variations including *ATM*, *ATR*, *BRCA1*, *BRCA2*, *PALB2*, *BRIP1*, *POLD1*, *RAD50*, *MLH1*, *MSH3*, *MSH6* and *PMS2*. Given an allelic fraction of 0.3 or higher, we counted 38 non-synonymous, four in-frame deletions as well as one insertion and one frameshift deletion. Thirty-five of the thirty-eight non-synonymous variants were listed as benign or likely benign in the ClinVar database. Six of the remaining nine mutations were seen in the *MSH3* gene. Three missense mutations, found in NC4, NC7 and ITAC3 (NM_002439: c.A2846G, p.Q949R), were identical and corresponded to a 0.8749 rate in normal tissue samples analyzed by Exome Aggregation Consortium (ExAC). The three NC6 patient-derived cell lines harbored an *MSH3* in-frame deletion (NM_002439:exon1:c.196_204del, p.P67_P69del). The *ATM* mutation in NC5 was a somatic mutation with an AF of 52% (NM_000051: c.A1597G, p.R533G). The somatic, heterozygotous *PMS2* frameshift deletion (AF of 50.7% in cancer genome) (NM_001322014: c.1239delA, p.D414Tfs*34) was supposed to cause a non-coding transcript variant. However, the NC5 showed a homozygous somatic *MLH1* missense mutation (AF 99.1%), leading to an exchange of aspartate to asparagine (NM_000249: c.G187A, p.D63N). The HR and MMR gene mRNA levels were analyzed in all cell lines, and no significant difference could be detected (Figure S1). The missense mutation found in *MLH1* of NC5 was already described to lead to an increased susceptibility of mutated protein to degradation [52]. 

In order to prove this hypothesis, protein expression patterns of the DNA MMR proteins MLH1, MSH2, MSH6 und PMS2 were analyzed via immunohistochemical staining in FFPE slides of cancer cell lines. Whereas the expression of the four MMR proteins remained stable in the NC1, NC4, NC6 (-primary,-LM,-relapse), NC7-NC9 and ITAC3, shown by an intensive nuclear positive staining reaction, a significantly reduced MLH1 staining and a complete loss of PMS2 expression was recorded for NC5, while MSH2 and MSH6 remained strongly expressed (Figure 2b). 

From this data, we concluded that NC5 was a highly microsatellite instable cell line (MSI-H) based on a somatic missense mutation in the ATB-binding site of MLH1, causing failure in complexing PMS2 and resulting in increased degradation of MLH1 and PMS2. 

### 3.3. MMR/MSI Analysis Elucidated further dMMR/MSI-H SNSCC 

**Figure 3:**
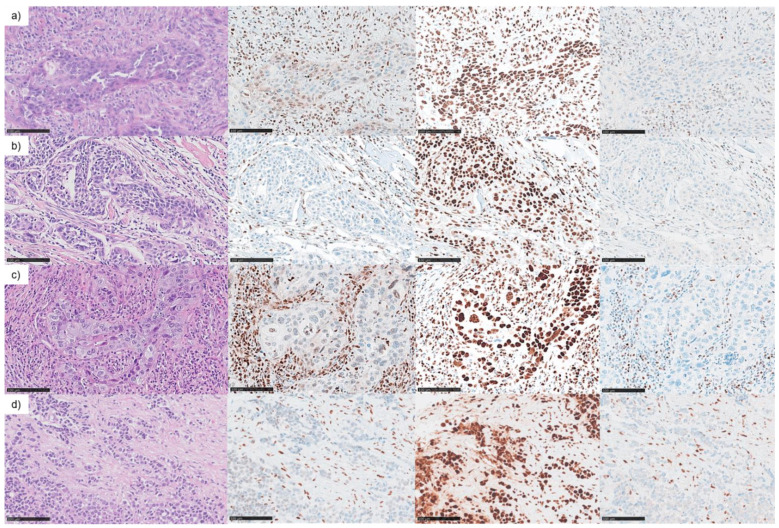
DNA mismatch repair deficiency (dMMR) in sinonasal squamous cell carcinoma (SNSCC). Immunohistochemical examination of DNA mismatch repair protein expression revealed four MMR deficient tumors by staining the loss of MLH1 and PMS2 in tumor cells compared to regular expression in tumor-associated/intratumoral immunocytes and regular expression of MSH6 and MSH2 (not shown) in tumor cells. (**a**) FFPE tumor sample referring to NC5 cell line of an 80-year-old man, (**b**) dMMR keratinizing SNSCC of a 53-year-old woman, (**c**) dMMR keratinizing SNSCC of a 70-year-old woman, (**d**) dMMR poorly differentiated non-keratinizing SNSCC of a 93-year-old woman. Left to right: H&E 400×, MLH1 400×, MSH6 400× and PMS2 400× (scale bars 100 µm).

In order to elucidate the MMR protein expression in a cohort of 220 sinonasal tumors, the four major components of the MMR complex, MLH1, MSH2, MSH6 and PMS2, were analyzed by IHC on the multitumor TMA and whole slide FFPE tumor samples. Of these, 174 samples of an adequate staining quality were assessed. The greatest histomorphological group of tumors were SNSCCs with 125 samples, and four (3.2%) showed a loss of PMS2 expression and at least a significantly reduced or complete loss of MLH1 expression in tumor cells. All four samples were tested negative for HPV. Histologically, three of these four SNSCCs were keratinising SNSCC with well to poor differentiation. One showed a poorly differentiated non-keratinizing histological phenotype (Figure 3). 

None of the other cancer types or SPs exhibited loss or significant reduction of MMR protein expression. In the 4 dMMR SNSCC, microsatellite instability was detected in the analyzed markers, which suggests that the reduction or loss of the MMR proteins MLH1 and PMS2 results in an MSI phenotype. One of these four cases was the primary tumor from which cell line (SCC)NC5 was derived. This tumor derived from an 80-year-old male. The NC5 primary tumor sample (Figure 3a) showed instability in four of five markers (instable BAT25, BAT26, D5S346 (APC) and D2S123 and stable D17S250 (MfD)). Two other cases showed instability in BAT26. The dMMR keratinizing SNSCC of a 70-year-old woman (Figure 3c) was additionally instable in BAT25, which led to MSI-H classification according to the Bethesda guidelines. The dMMR keratinizing SNSCC of a 53-year-old woman (Figure 3b) had to be classified as MSI-L according to Bethesda guidelines. A slightly heterogeneous MLH1 staining reaction was observed in the MSI-L carcinoma, especially in the early invasive growing parts of the tumor, which could have been an indication of residual activity of the MMR system in some tumor parts. In addition, this staining pattern rather indicated a secondary occurrence of MLH1 dysfunction in this tumor. The convincing loss of immunodetectability of MSH1 und PMS2 in deeper invasive tumor parts with increased intratumoral immunocytes suggested a MSI phenotype. The dMMR poorly differentiated non-keratinizing SNSCC of a 93-year-old woman (Figure 3d) could not be investigated further due to the reduced sample size and DNA quality.

### 3.4. Targeted Panel Analysis of Cancer Biomarkers in Sinonasal Tumors

**Figure 4:**
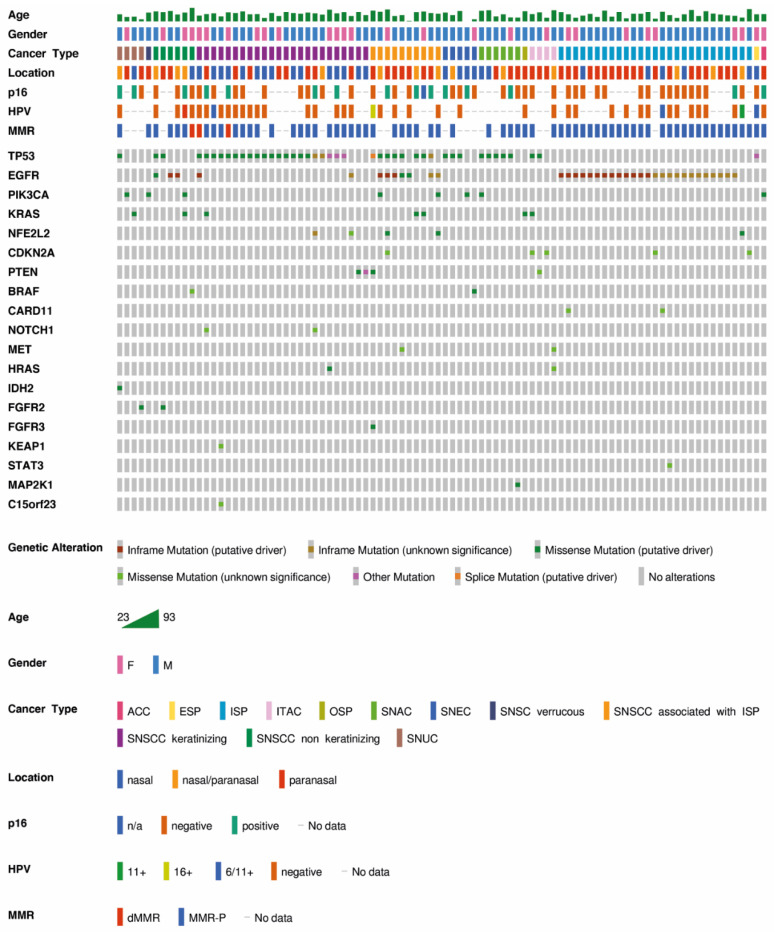
Next generation sequencing (NGS) results of a sinonasal carcinoma collective. A total of 136 FFPE samples from sinonasal tumors, from 90 male and 46 female individuals, were analyzed by next generation-targeted sequencing. Age at diagnosis ranged from 23 to 93 years (mean age 61 years, SD 13 years) (cases that were WT in all tested gene regions were not shown). *EGFR* mutations were significantly associated with ISP and SP-associated carcinomas, whereas *TP53* mutations could be detected in many different tumor types. In contrast to *PIK3CA* mutations, *KRAS* and *EGFR* mutations were mutually exclusive. HPV positive cases predominantly carried a WT sequence in the *EGFR* gene.

Given that sinonasal tumors are rare tumors with a poor prognosis, we further analyzed a collective of sinonasal tumors regarding clinically actionable driver mutations, including mutations detected by cell line WES and typical drug-targetable mutations. In addition to keratinizing and non-keratinizing SNSCC, we analyzed SNSCC associated with ISP as well as sarcomatoid and verrucous SNSCC. Although a precancerous lesion, we included sinonasal papillomas (SPs), as they are often seen in association with invasive carcinoma and can be difficult to distinguish from well-differentiated SNSCC. Furthermore, we added adenocarcinomas of different subtypes (ITAC, SNAC and ACC), as well as SNEC and SNUC, as they are a differential diagnosis of poorly differentiated SNSCC. In total, 136 sinonasal tumors were sequenced by a next generation sequencing (NGS) panel analysis. (Figure 4). In total, 136 genetic alterations were detected in 90 individual cases with a maximum of four mutations per tumor sample (Table S3). A total of 46 of 136 (34%) tumors showed wild type (WT) sequences in all analyzed genes. Mutational frequency rate in the histologically different tumor samples was determined (Table S4). 

The *EGFR* mutation correlated significantly with the ISP tumor histotype (*p* < 0.001). A total of 89.3% (25/28) of the ISPs carried *EGFR* exon 20 mutations, which corresponded to 73.5% of *EGFR* mutations in the entire cohort. The *EGFR* exon 20 mutations clustered between amino acid positions 762 and 774. The most prevalent recurrent mutation was p.N771_H773dup (28%, 7/25). *TP53* and *EGFR* mutation occurred in a mutually exclusive pattern in ISPs, whereas the ISP-associated carcinomas harbored simultaneously an ISP-typical *EGFR* exon 20 mutation and a *TP53* mutation in 40% of cases (4/10). One ISP associated SNSCC without a *TP53* mutation had an *EGFR* mutation combined with *PIK3CA* and *NFE2L2* mutation. A total of 40% of *TP53* mutated the ISP-associated SNSCC (4/10) without an *EGFR* mutation and harbored additional *HRAS*, *PTEN* or *KRAS* mutations. For one case of an ISP-associated SNSCC, only an *EGFR* exon 20 mutation was reported. In this case, a *TP53* and *PIK3CA* mutation were detectable below the AF cut-off of 5%. In addition, a part of SNSCC non- and keratinizing with no documented ISP simultaneously to carcinoma or in patient’s history showed *EGFR* mutations, while none of the other carcinoma types were *EGFR* mutated*,* indicating a common origin of tumors with the *EGFR* mutation. The *EGFR* and *KRAS* mutation were mutually exclusive. A *KRAS* mutation (p.G12V) was detected in a case of oncocytic sinonasal papilloma (OSP), which emphasized that *EGFR* and *KRAS* mutations were independent early-occurring driver mutations. While in ITAC an identical *KRAS* (p.G12V) was detected, an SNSCC non-keratinizing and SNSCC-associated with an ISP carried a *KRAS* gene alteration leading to an exchange of glycin to aspartic acid (p.G12D). The *KRAS* mutation in SNUC affected the NKxD nucleotide binding motif (p.D119N). *IDH2* mutations were exclusively found in SNUCs, combined with a *TP53* or a *PIK3CA* mutation.

Overall, the rate of *TP53* mutated tumors was 30.9% (42/136). Six samples exhibited more than one *TP53* mutation. Mutational hotspots could not be detected. TP53 gene alterations were frequently found in exon 5 (32%, 16/50) and exon 6 (30%, 15/50). SPs were *TP53* wild type, except for one ESP, which was positive for low-risk HPV (type 6/11+) and carried an in-frame mutation in*TP53* gene, of unknown significance. 

*PIK3CA* was mutated in different tumor types (SNSCC, SNEC, SNUC, SNAC and ACC). *CDKN2A* mutations were found in an ISP-associated SNSCC, and an ISP as well as in ITAC combined with *KRAS, EGFR* and or *TP53* mutations. Putative oncogenic and targetable mutations could be found in individual tumor samples in *FGFR2* (SNUC, SNSCC non-keratinizing), *FGFR3* (SNSCC-associated with ISP), *MAP1K2* (SNAC), *MET* (ISP-associated SNSCC), *MAP2K1* (SNAC) and *HRAS* (SNSCC keratinizing and ITAC).

Due to the finding of a putative oncogenic *CARD11* mutation in SNSCC cell lines and the fact that *CARD11* mutations were reported in HNSCC with a frequency of 5.8%, we further included *CARD11* exons in a newly designed NGS-panel (Table S2). Mutations were exclusively found together with *EGFR* mutations in ISPs. *NOTCH1* mutations were recurrently reported in HNSCC (17.1% HNSCC, TCGA); in our cohort we could only confirm two mutations in keratinized SNSCC. NRF2 (*NFE2L2*) and KEAP1 (*KEAP1*), key regulators of oxidative stress, were mutated in a subset of HNSCC (TCGA data *NFE2L2* 5.4% and *KEAP1* 4.1%). *NFE2L2* mutations were found in one ISP, two ISP-associated SNSCC and one keratinized SNSCC, whereas *KEAP1* mutations only accrued in one keratinized SNSCC. HPV-positive tumors made up only a small proportion and correlated significantly with the histological phenotype of tumors with squamous cell differentiation (*p* < 0.001) in our tumor population, which may be underrepresented, as not all cases could be tested for HPV. In the literature, up to 31.5% of SNSCC are HPV positive [53], and HPV is more commonly found in sinonasal subsites with increased exposure to refluxed oropharyngeal secretions and in geographic regions where HPV+ oropharyngeal squamous cell carcinoma (OPSCC) is more prevalent [54]. In our cohort, the frequency of HPV high-risk infection was 5.25% in all tested cases (3/57), and only one exophytic sinonasal papilloma (ESP) (16.7%,1/6), one ISP-associated carcinoma (10%, 1/10) and one SNSCC non-keratinizing (7.7%, 1/13) were affected. Low-risk types of HPV-infection were detected in ISPs, ESP and keratinizing SNSCC in 10.5% of all tested cases (6/57). One HPV-positive ISP showed an additional *EGFR* mutation. 

The mutational profiles of the four dMMR/MSI cancers were heterogenic. One case was wildtype in all tested genes. Two dMMR/MSI tumors showed *TP53* mutations, one of which in combination with the *EGFR* exon 20 mutation, which suggests that deficiency in the MMR system could be both an early or late event in tumorigenesis.

In summary, therapeutically targetable *EGFR* exon 20 mutations are a hallmark of ISP and ISP-associated SNSCC. Oncogenic *KRAS* mutations were found in the absence of *EGFR* mutations in different histological cancer subtypes and in one case of an OSP. While we found dMMR/MSI SNSCC at a frequency of 3.2% (4/125), none of the other sinonasal cancer types or SPs showed a dMMR phenotype. Potential driver mutations in *EGFR* and dMMR/MSI were not mutually exclusive. The relation between driver mutations and dMMR/MSI status regarding their impact on prediction of sensitivity to targeted therapies and clinical outcome needs to be evaluated clinically. 

## 4. Discussion

Sinonasal carcinomas are rare tumors with an overall poor prognosis. Due to the location near the central nervous system and due to their frequent diagnosis in advanced stages, local therapeutic approaches are limited, and systemic therapeutic options are needed. While the treatment of advanced head and neck squamous cell carcinoma (HNSCC) has developed from platinum-based chemotherapy to molecular targeted therapy with agents, such as cetuximab (anti-EGFR antibody) and PD-1/PD-L1 Immune Checkpoint Inhibitors (ICIs) [55,56,57], sinonasal carcinomas are missing in global clinical trials [55,56,57]. PD-1/PD-L1 ICIs have been shown to provide therapeutic advantages for recurrent or metastatic HNSCC cases, as compared to standard chemotherapies [55,56,57]; the combination of cetuximab with PD-1 ICIs provides promising clinical data [58]. FDA-approved biomarker dMMR or MSI-H for PD-1/PD-L1 ICI treatment decision for tumors regardless of their primary site of origin [35], however, has not yet been comprehensively investigated in sinonasal carcinomas.

In our study, we demonstrated the existence of dMMR/MSI-H sinonasal tumors by testing MMR protein expression in multitumor TMA and in corresponding whole slide tumor staining of 174 tumor samples, including the most common tumor types of this area: SNSCC, adenocarcinoma, SNEC, SNUC and SPs. Only SNSCC were affected with a frequency of 3.2% (4/125), while all analyzed sinonasal adenocarcinoma types (ACC, SNAC and ITAC) as well as SNUC and SNEC displayed intact MMR protein expression patterns; SPs such as ISP, ESP and OSP also displayed a pMMR phenotype. The dMMR tumors showed a reduction or even loss of MLH1 expression combined with a loss in PMS2. MLH1 expression was slightly heterogenic, especially in early invasive growing parts of the tumor. This phenomenon is already known and refers to an underlying mechanistic defect in the MMR system [59]. *MLH1* missense mutation and *MLH1* promoter methylation might result in a weakly detectable but functionally insufficient protein [59]. Based on this observation, the four MMR proteins should be carefully assessed. MSI testing according to Bethesda guidelines of dMMR tumors and pMMR controls revealed concordant results. It should be mentioned that MSI testing using the Bethesda panel was initially established for detection of tumors belonging to the spectrum of Lynch syndrome [60]. In sinonasal carcinomas, therefore, a comparative assessment of microsatellite regions in matched normal tissue controls is mandatory and should be assessed in alignment with MMR-IHC. More studies are needed to evaluate the assignability of the Bethesda panel for MSI testing in sinonasal cancers and to prove the impact of other microsatellite regions in the different histological subtypes. In areas where reduced MLH1 expression is detectable by immunhistochemistry, MSI analysis can clarify an ambiguous result (MSI-L). Tumor areas for MSI examination should be determined according to the MMR staining results. Mechanistically, we identified a homozygous somatic missense mutation in the ATPase region of *MLH1* in one keratinizing SNSCC cell line of an 80-year-old man when analyzing 10 sinonasal cancer cell lines by WES. This mutation was already described to result in rapid degradation of the mutated protein [52]. The accompanying loss of PMS2 can be explained by secondary degradation of the protein due to the MLH1 deficiency. This phenomenon was shown in colon cancers for MLH1 deficiency based on promotor methylation and *MLH1* mutations [61,62]. The additional heterozygous *PMS2* frameshift mutation is most likely a secondary event of pre-existing MMR deficiency according to MLH1 deficiency. Analysis of MMR gene expression revealed an unaffected mRNA level in all cancer cell lines, and an MLH1 promoter methylation was excluded. 

So far, only a few studies have addressed the MSI/MMR status in sinonasal carcinomas, with a resulting frequency of MSI for ITACs of 2% and between 2–21% in dMMR/MSI for SNSCCs [63,64,65,66]. Although dMMR/MSI-H SNSCCs are a small subgroup of SNSCC, they are clearly molecularly defined and they are most likely sensitive to ICIs. For that reason, the MMR-IHC in combination with MSI testing should be included in the diagnostic workflow of SNSCC to identify biomarkers for ICIs treatment decision. 

This study retrospectively examined sinonasal tumors from two European hospitals over the past three decades and was limited due to the fact that the groups of different histological subtypes contained a small sample size and to the missing clinical correlation. 

Apart from MSI analysis, our targeted-NGS panel analysis of 136 sinonasal carcinoma and WES of the 10 patient-derived cell lines showed, as expected from previous studies [19,20,25], that *EGFR* exon 20 mutations (EGFRex20ins) are a hallmark of ISPs. Cases of SNSCC with simultaneous ISP or with ISP in the patient’s history showed an *EGFR* mutation in 50% (5/10) of cases and further an inactivating *TP53* missense mutation, as published before [25]. Overall frequency of *EGFR* mutations in SNSCC was 41.9% (13/31). The main mutation type was EGFRex20ins. The EGFRex20ins were clustered between amino acid positions 762 and 774, similar to a known molecular subgroup of pulmonary adenocarcinoma [67]. The most prevalent recurrent mutation was p.N771_H773dup (10/37) in ISPs and SNSCC. Based on extended research in lung adenocarcinoma, it is current knowledge that almost all EGFRex20ins confer in vitro and clinical resistance to first- and second-generation EGFR tyrosine kinase inhibitors (TKIs) [67,68], although osimertinib had shown clinical efficacy against some of these mutations [69,70]. Amivantamab (amivantamab-vmjw; Rybrevant™), a bispecific monoclonal antibody [71], and Mobocertinib (TAK 788), a pan-mutation-selective irreversible EGFR TKI [72], are promising targeted agents to overcome the EGFRex20ins-mutation resistance to EGFR TKIs of previous generations and offer new treatment regimens for EGFRex20ins-mutated SNSCCs. It should be taken into account that the dMMR phenotype and EGFRex20ins-mutated status in SNSCC are not exclusive. This phenomenon should be further investigated regarding its frequency and biological effect and has to be considered in planning the best therapeutic option. 

In our study, the overall incidence of the *EGFR* mutant SNSCC was 41.9%, which is in line with previous studies reporting *EGFR* mutations in 15–50% [19,20,25], indicating a promising therapeutic option with next-generation EGFR TKI and should encourage prospective clinical trials. 

Intestinal-type *KRAS* mutations p.G12V were found in OSP and ITAC as well as *KRAS* p.G12D mutations in SNSCC. While G12C is the most prevalent *KRAS* mutation, G12D is found in up to 3% in lung adenocarcinoma and is suspected to define a special subtype of *KRAS/TP53* mutant tumors with low TMB, reduced PD-L1 expression and immune cell infiltration, so that this co-mutation status might be a negative predictive biomarker for PD-1 ICIs [73]. Previously published data showed an overall mutation frequency of common *KRAS* mutations of 100% in OSPs (n = 51) [74], and mixed-types of OSP and ISPs were already reported [66]. Two molecularly different Papilloma-Carcinoma-sequence could be hypothesized, either driven by *EGFR* or *KRAS* mutations and a secondary event as, for example, a *TP53* mutation leading to invasive carcinoma. 

In addition, potentially targetable mutations in *PIK3CA, FGFR2, FGFR3, BRAF, HRAS, MAP2K1, PTEN*, and *NOTCH1* were found and could be associated with progressive disease [75], but further analyses are needed in sinonasal carcinomas. While *CARD11* mutations, which lead to a constitutively activated NFκB pathway, were demonstrated to drive diffuse large B-cell lymphoma (DLBCL) pathogenesis [50], the role in sinonasal carcinoma is unclear. Through a WES analysis, we found *CARD11* mutations in two cell lines; one of them was suspected to affect the coil-coiled domain of the protein indicating an activating effect on NFκB signalling pathway. In our cohort of sinonasal tumors, *CARD11* mutations were detected in combination with an *EGFR* mutation in two analyzed ISP. Although *CARD11* mutations were already described in HNSCC [51] and cutaneous squamous cell carcinoma (SCC) [34], the role in the pathogenesis of SNSCC was not yet sufficiently investigated. Further analyses are required to show if this mutation results in a constitutive activation of the NFκB signaling pathway, which harbors the possibility of a treatment with NFκB inhibitors [76].

## 5. Conclusions

In conclusion, we comprehensively analyzed and characterized sinonasal tumors based on their histomorphological characteristics and molecular properties in a cohort of 220 sinonasal tumors and 10 corresponding sinonasal cancer cell lines. The molecular subclassification, including immunhistochemical and sequencing-based diagnostic approaches, provides useful information for selecting an individualized therapeutic strategy. Besides the promising and therapeutically targetable *EGFR* mutant SNSCC subgroup, we confirm a dMMR/MSI subtype of SNSCC, which may confer clinical benefit to ICI treatment. 

## Data Availability

Data can be obtained upon reasonable request to the corresponding author.

## Supplementary Materials

The following are available online at https://www.mdpi.com/article/10.3390/cancers13236081/s1: Table S1: Tumor samples and analyzes, Table S2: Newly designed targeted NGS panel, Table S3: Targeted panel sequencing result of sinonasal tumor cohort, Table S4: Mutation frequency in sinonasal tumor cohort, Figure S1: mRNA expression of DNA mismatch repair and homologous repair (HR) genes.
