# Supplementary material for: Mismatch Repair Deficiency and Somatic Mutations in Human Sinonasal Tumors
Source: Cancers (Basel). 2021 Dec 2;13(23):6081. doi: 10.3390/cancers13236081 (PMC8657279; doi:10.3390/cancers13236081)
Supplement: Supplementary file 1 [file cancers-13-06081-s001.zip › Table S1_Tumor samples and analyzes.pdf]

Table S1: Tumor samples and analyzes

		Institution			Gender						Location			MMR-		Tageted Panel Analysis	
		FFPE Cologne	FFPE Oviedo	Slides Oviedo	male			female			para-nasal	nasal	para-nasal and nasal	MMR-analysed	N/A	WT samples	mutated sampels
		N	N	N	N	Age Mean	Age SD	N	Age Mean	Age SD	N	N	N	N	N	N	N
Subtype	SNSCC keratinizing	43	23	41	69	65	10	38	61	12	64	42	1	99	8	7	24
	SNSCC non-keratinizing	10	2	1	6	64	14	7	65	17	6	5	2	12	1	2	6
	SNSCC associated with ISP	10	1	1	8	61	17	4	79	6	8	1	3	9	3	0	10
	SNSCC sarcomatoid	4	0	0	3	60	12	1	70		1	3	0	3	1	3	0
	SNSC verrucous	2	0	0	2	69	0	0			2	0	0	2	0	0	1
	ISP	28	0	0	21	63	12	7	49	13	18	7	3	23	5	1	27
	ESP	6	0	0	3	52	8	3	43	15	2	4	0	4	2	5	1
	OSP	1	0	0	1	76		0			1	0	0	1	0	0	1
	ACC	11	0	0	5	66	12	6	63	12	5	2	4	6	5	10	1
	SNAC	11	0	0	8	66	16	3	56	11	6	4	1	6	5	5	6
	ITAC	7	0	0	6	69	8	1	42		3	2	2	3	4	2	4
	SNUC	6	4	0	6	56	14	4	52	9	4	3	3	4	6	7	4
	SNEC	2	6	0	7	54	19	1	34		2	4	2	2	6	4	5
	Total N	141	36	43	145	820	142	75	613	95	122	77	21	174	46	46	90

FFPE = formalin-fixed and paraffin embedded, N = sample number, MMR = mismatch repair, WT = wild type, SNSCC = sinonasal squamous cell carcinoma, ISP = inverted sinonasal papilloma, ESP = exophytic sinonasal papilloma, OSP = oncocytic sinonasal papilloma, ACC = adenoid cystic carcinoma, SNAC = adenocarcinoma with no intestinal nor salivary gland differentiation, ITAC = intestinal type adenocarcinoma SNEC = sinonasal neuroendocrine carcinoma, sinonasal undifferentiated carcinoma = SNUC
