# Supplementary material for: Mismatch Repair Deficiency and Somatic Mutations in Human Sinonasal Tumors
Source: Cancers (Basel). 2021 Dec 2;13(23):6081. doi: 10.3390/cancers13236081 (PMC8657279; doi:10.3390/cancers13236081)
Supplement: Supplementary file 1 [file cancers-13-06081-s001.zip › Table S3_Targeted panel sequencing result of sinonasal tumor cohort.pdf]

Table S3_Targeted panel sequencing result of sinonasal tumor cohort

**sample n°**	**gender**	**histotype**	**age at diagnosis**	**location**	**p16**	**HPV**	**MMR/MSI**	**gene name**	DNA-sequencechange	aminoacidchange
1	M	SNEC	77	nasal	negative	negative	N/A	TP53	c.517G>T	p.V173L
2	M	SNUC	54	paranasal	positive	N/A	N/A	WT		
3	M	SNSCC associated with ISP	63	nasal/paranasal	N/A	negative	MMR-P	EGFR	c.2310_2311insGGGTTG	p.N771delinsGLN
								NFE2L2	c.241G>A	p.G81S
								PIK3CA	c.1633G>A	p.E545K
4	F	SNSCC non keratinizing	70	paranasal	N/A	N/A	MMR-P	WT		
5	M	SNSCC keratinizing	58	nasal/paranasal	negative	N/A	N/A	TP53	c.648_649insTGTG	p.V217Cfs*6
6	M	SNSCC keratinizing	69	paranasal	negative	negative	MMR-P	TP53	c.844C>T	p.R282W
7	F	ISP	42	paranasal	negative	negative	MMR-P	EGFR	c.2303_2311dup	p.S768_D770dup
8	M	SNSCC keratinizing	63	nasal	negative	N/A	MMR-P	TP53	c.637C>T	p.R213*
9	F	ACC	50	nasal	N/A	N/A	N/A	WT		
10	M	SNSCC keratinizing	66	nasal	N/A	N/A	MMR-P	TP53	c.824G>A	p.C275Y
11	F	SNSCC associated with ISP	85	paranasal	positive	N/A	N/A	CDKN2A	c.442G>A	p.A148T
								EGFR	c.2303_2311dup	p.S768_D770dup
								NFE2L2	c.83T>C	p.I28T
								TP53	c.404G>A	p.C135Y
12	M	SNSCC keratinizing	72	nasal	N/A	N/A	MMR-P	PTEN	c.494G>A	p.G165E
13	M	SNSCC non keratinizing	59	nasal/paranasal	negative	negative	MMR-P	EGFR	c.2303_2311dup	p.S768_D770dup
14	F	ISP	72	nasal	negative	negative	MMR-P	EGFR	c.2311_2319dup	p.N771_H773dup
15	M	ACC	77	paranasal	negative	N/A	MMR-P	WT		
16	M	SNAC	60	paranasal	N/A	N/A	MMR-P	WT		
17	F	SNSCC keratinizing	51	paranasal	negative	negative	MMR-P	EGFR	c.2311delinsGTT	p.N771delinsGY
								NOTCH1	c.80A>G	p.D27G
18	M	ESP	62	nasal	negative	6/11+	MMR-P	WT		
19	M	SNAC	76	paranasal	positive	N/A	MMR-P	WT		
20	M	ISP	63	paranasal	negative	negative	MMR-P	EGFR	c.2311_2319dup	p.N771_H773dup
21	M	SNSCC keratinizing	72	paranasal	N/A	N/A	N/A	TP53	c.814G>C	p.V272L
22	F	SNSCC keratinizing	51	paranasal	N/A	N/A	N/A	TP53	c.517G>T	p.V173L
23	M	SNSCC keratinizing	65	paranasal	positive	negative	MMR-P	NFE2L2	c.60_62dup	p.D21dup
								NOTCH1	c.1154C>T	p.S385F
								TP53	c.638delG	p.R213Hfs*34
24	M	SNSCC keratinizing	49	nasal	N/A	negative	MMR-P	TP53	c.560_573del	p.G187Afs*17
								KEAP1	c.1727C>T	p.T576M
								C15orf23	c.140C>A	p.T47K
								TP53	c.770T>C	p.L257P
25	M	SNSCC non keratinizing	77	paranasal	N/A	N/A	MMR-P	EGFR	c.2303_2311dup	p.S768_D770dup
26	M	SNSCC keratinizing	56	nasal	N/A	N/A	MMR-P	PTEN	c.640C>T	p.Q214*
								TP53*	c.584T>G	p.I195S
								PIK3CA*	c.1634A>C	p.E545A

27	F	ISP	52	paranasal	positiv	negative	MMR-P	EGFR	c.2304_2305insATGGACAGC	p.S768_V769insMDS
28	F	SNSCC keratinizing	71	nasal	N/A	negative	MMR-P	TP53	c.376-2A>G	
29	F	SNSCC keratinizing	54	nasal	N/A	N/A	MMR-P	WT		
30	M	SNSCC keratinizing	65	nasal	negative	6/11+	MMR-P	TP53	c.428T>A	p.V143E
31	F	SNAC	62	nasal	negative	N/A	MMR-P	WT		
32	M	SNSCC associated with ISP	79	paranasal	negative	16+	MMR-P	TP53	c.559+3G>T	c.559+3G>T
								FGFR3	c.746C>G	p.S249C
								PTEN	c.277C>T	p.H93Y
33	M	SNAC	78	nasal	N/A	N/A	N/A	PIK3CA	c.3140A>T	p.H1047L
								TP53	c.743G>A	p.R248Q
34	M	SNSCC associated with ISP	56	paranasal	N/A	N/A	MMR-P	TP53	c.647T>G	p.V216G
								EGFR	c.829_830delinsGA	p.M277E
								MET	c.3029C>T	p.T1010I
35	F	SNSCC keratinizing	84	nasal	N/A	N/A	MMR-P	HRAS	c.182_183AG>TT	p.Q61L
								TP53	c.673-2A>G	
36	M	SNSCC keratinizing	61	nasal	N/A	N/A	MMR-P	WT		
37	M	SNSC verrucous	69	paranasal	N/A	N/A	MMR-P	PIK3CA	c.1633G>A	p.E545*
38	F	SNSCC keratinizing	50	nasal	N/A	negative	MMR-P	WT		
39	F	ACC	54	nasal/paranasal	negative	N/A	MMR-P	WT		
40	M	ISP	54	paranasal	N/A	N/A	MMR-P	EGFR	c.2310_2324dup	p.N771_C775dup
41	M	ISP	63	paranasal	N/A	N/A	MMR-P	EGFR	c.2303_2311dup	p.S768_D770dup
42	M	ITAC	81	nasal/paranasal	negative	negative	MMR-P	MET	c.3415G>A	p.V1139I
								HRAS	c.400G>A	p.A134T
43	F	SNAC	44	paranasal	positive	N/A	MMR-P	TP53	c.430C>T	p.Q144*
44	M	SNSCC associated with ISP	73	nasal/paranasal	N/A	negative	N/A	EGFR	c.2301_2309dup	p.S768_D770dup
								PIK3CA	c.1633G>A	p.E545K
								TP53	c.844C>T	p.R282W
45	F	SNSCC keratinizing	55	nasal	N/A	N/A	MMR-P	WT		
46	F	ISP	37	paranasal	N/A	N/A	N/A	CDKN2A	c.442G>A	p.A148T
								EGFR	c.2332C>G	p.L778V
								EGFR	c.2296_2297insTGGCCAGCG	p.M766delins
47	M	ITAC	61	paranasal	negative	N/A	MMR-P	KRAS	c.35G>T	p.G12V
								CDKN2A	c.442G>A	p.A148T
								TP53	c.614A>G	p.Y205C
48	M	ISP	59	paranasal	N/A	N/A	MMR-P	EGFR	c.2302_2305delinsCCGC	p.S768_V769delinsPL(25
49	M	SNSCC sarcomatoid	74	nasal	N/A	negative	MMR-P	WT		
50	M	SNAC	60	nasal/paranasal	negative	N/A	MMR-P	TP53	c.524G>A	p.R175H
51	M	SNSCC keratinizing	67	nasal	N/A	N/A	MMR-P	TP53	c.527G>A	p.C176Y
52	M	ISP	87	nasal	N/A	N/A	MMR-P	CDKN2A	c.442G>A	p.A148T
53	M	SNAC	49		N/A	N/A	N/A	TP53	c.403T>C	p.C135R34
54	F	ISP	39	paranasal	N/A	negative	MMR-P	EGFR	c.2311_2319dup	p.N771_H773dup
55	F	SNSCC keratinizing	51	nasal	negative	negative	N/A	TP53	c.309C>G	p.Y103*

56	F	SNUC	64	nasal/paranasal	negative	N/A	MMR-P	WT		
57	M	ITAC	60		N/A	N/A	N/A	WT		
58	F	SNUC	51	nasal/paranasal	negative	negative	MMR-P	WT		
59	M	ISP	64	nasal/paranasal	N/A	N/A	MMR-P	EGFR	c.2310_2311insGGG	p.N771delinsGF
60	F	ACC	55	paranasal	negative	N/A	MMR-P	WT		
61	M	ISP	72	paranasal	N/A	N/A	MMR-P	EGFR	c.2308_2316dup	p.D770_P772dup(12
62	F	ACC	77	nasal	positive	N/A	MMR-P	WT		
63	M	ACC	54	paranasal	N/A	N/A	N/A	WT		
64	F	ACC	79	nasal/paranasal	N/A	N/A	N/A	WT		
65	M	SNUC	64	paranasal	negative	N/A	MMR-P	WT		
66	M	ISP	76	paranasal	negative	negative	N/A	EGFR	c.2311delinsGGT	p.N771delinsGY
67	M	ISP	48	paranasal	N/A	negative	MMR-P	EGFR	c.2311_2319dup	p.N771_H773dup
68	M	ISP	56	paranasal	N/A	negative	MMR-P	WT		
69	F	SNSCC non keratinizing	69	nasal/paranasal	positive	16+	MMR-P	KRAS	c.35G>A	p.G12D
								PIK3CA	c.1132T>C	p.C378R
70	F	ESP	60	nasal	negative	6/11+	MMR-P	TP53	c.757_758insA	p.T253Nfs*11
71	M	ESP	48	nasal	negative	58+	MMR-P	WT		
72	M	ISP	79	paranasal	negative	N/A	MMR-P	EGFR	c.2311_2319dup	p.N771_H773dup
73	M	SNUC	60	nasal/paranasal	positive	negative	MMR-P	IDH2	c.515G>C	p.R172T
								TP53	c.743G>A	p.R248Q
74	F	SNSCC keratinizing	61	paranasal	positive	negative	MMR-P	KRAS	c.35G>T	p.G12V
								NOTCH1	c.1030T>A	p.C344S
								TP53	c.742C>T	p.R248W
75	M	ISP	51	paranasal	negative	negative	MMR-P	EGFR	c.2313_2314insGTC	p.N771_P772insV
76	M	ACC	74	nasal/paranasal	negative	N/A	MMR-P	WT		
77	F	ISP	60	paranasal	negative	negative	MMR-P	EGFR	c.2311_2319dup	p.N771_H773dup
								CARD11	c.2920C>T	p.R974C
78	F	SNSCC keratinizing	61	nasal	N/A	negative	MMR-P	TP53	c.309C>G	p.Y103*
79	M	ISP	56	paranasal	negative	negative	MMR-P	EGFR	c.2236_2253del	p.E746_T751del
79		ISP			N/A	N/A	N/A	STAT3	c.2050G>A	p.G684R
80	M	OSP	76	paranasal	negative	negative	MMR-P	KRAS	c.35G>T	p.G12V
81	M	SNSCC keratinizing	60	paranasal	N/A	negative	MMR-P	TP53	c.742C>T	p.R248W
82	F	SNSCC sarcomatoid	70	paranasal	N/A	negative	MMR-P	WT		
83	M	SNSCC keratinizing	75	nasal	negative	negative	MMR-P	TP53	c.747G>T	p.R249S
84	M	ITAC	66	nasal	N/A	N/A	N/A	WT		
85	M	ISP	73	nasal	negative	6/11+	MMR-P	CARD11	c.743A>G	p.N248S
85		ISP			N/A	N/A	N/A	EGFR	c.2304_2305insATGGACAGC	p.S768_V769insMDS
86	M	SNAC	81	nasal	N/A	N/A	N/A	TP53	c.314G>T	p.G105V(64
87	M	SNSCC non keratinizing	53	paranasal	negative	negative	MMR-P	WT		
88	M	ISP	46	paranasal	negative	N/A	MMR-P	EGFR	c.2311_2319dup	p.N771_H773dup
89	F	SNSCC keratinizing	65	nasal	positive	negative	N/A	TP53	c.783-24_782del	
90	M	ISP	82	paranasal	negative	negative	N/A	EGFR	c.2313_2314insGTG	p.N771_P772insV

91	M	SNSCC associated with ISP	51	paranasal	negative	negative	MMR-P	TP53	c.844C>T	p.R282W
								EGFR	c.2303_2311dup	p.S768_D770dup
								EGFR	c.743G>A	p.R248Q
								TP53	c.584T>G	p.I195S
92	M	SNSCC associated with ISP	26	paranasal	negative	negative	MMR-P	EGFR	c.2311_2312insCAC	p.N771delinsTH
93	M	SNEC	56	nasal/paranasal	negative	N/A	MMR-P	TP53	c.404G>C	p.C135S
								TP53	c.844C>T	p.R282W
94	F	ISP	43	nasal/paranasal	negative	11+	MMR-P	NFE2L2	c.95T>G	p.V32G
95	F	SNSCC keratinizing	60	nasal	N/A	negative	MMR-P	WT		
96	M	ISP	62		N/A	N/A	N/A	EGFR	c.2311_2319dup	p.N771_H773dup
97	M	ISP	71	paranasal	N/A	N/A	N/A	EGFR	c.2311_2319dup	p.N771_H773dup
98	M	ACC	72	nasal/paranasal	N/A	N/A	N/A	WT		
99	F	SNSCC non keratinizing	93	nasal	negative	negative	dMMR	BRAF	c.1363G>T	p.G455W
100	F	ACC	61	paranasal	positive	negative	MMR-P	PIK3CA	c.1636C>A	p.Q546K
101	M	SNEC	72	nasal	positive	N/A	MMR-P	WT		
102	M	ESP	47	nasal	N/A	6/11+	MMR-P	WT		
103	M	ACC	51	paranasal	N/A	N/A	N/A	WT		
104	F	SNEC	34	nasal	negative	N/A	N/A	BRAF	c.1781A>G	p.D594G
105	M	SNUC	34	paranasal	negative	N/A	N/A	FGFR2	c.2032A>G	p.R678G
106	M	SNUC	48	nasal	positive	N/A	N/A	KRAS	c.355G>A	p.D119N
107	F	SNUC	46	paranasal	positive	N/A	N/A	WT		
108	M	SNUC	75	nasal	negative	N/A	N/A	WT		
109	M	SNEC	69	paranasal	negative	N/A	N/A	WT		
110	M	SNEC	56	nasal/paranasal	x	N/A	N/A	WT		
111	M	SNEC	33	paranasal	positive	N/A	N/A	WT		
112	M	SNEC	23	nasal	positive	N/A	N/A	PIK3CA	c.3140A>G	p.H1047R
113	M	SNEC	69	nasal	positive	N/A	N/A	TP53	c.743G>A	p.R248Q
114	M	SNUC	54		n.a.	N/A	N/A	WT		
115	M	ISP	60	nasal	negative	N/A	MMR-P	EGFR	c.2316_2321dup	p.H773_V774dup
116	F	SNSCC associated with ISP	74	paranasal	n/a	N/A	N/A	TP53	c.534C>A	p.H178Q
								TP53	c.537delT	p.H179Qfs*68
								KRAS	c.35G>A	p.G12D
117	F	ESP	38	paranasal	n/a	negative	N/A	WT		
119	F	ITAC	42	paranasal	N/A	N/A	N/A	CDKN2A	c.442G>A	p.A148T
120	F	SNUC	46	paranasal	N/A	N/A	N/A	PIK3CA	c.3140A>G	p.H1047R
121	M	SNSCC keratinizing	52	paranasal	N/A	N/A	N/A	WT		
122	M	SNSCC associated with ISP	71	nasal/paranasal	positive	N/A	MMR-P	KRAS	c.35G>A	p.G12D(58
								TP53	c.743G>A	p.R248Q(70
123	M	SNSCC associated with ISP	66	paranasal	positive	N/A	MMR-P	TP53	c.672+1G>T	Substitution - intronic
								EGFR	c.2316_2317insAACAACCCC	p.P772_H773insNNP
124	M	ITAC	70	paranasal	N/A	N/A	N/A	TP53	c.847C>T	p.R283C
								TP53	c.711G>C	p.M237I

								PTEN	c.1061C>A	p.P354Q
								TP53	c.775G>C	p.D259H
125	F	SNSCC non keratinizing	71		N/A	N/A	N/A	FGFR2	c.1693G>A	p.E565K
126	F	ESP	31		N/A	N/A	N/A	WT		
127	F	SNAC	63	paranasal	N/A	N/A	N/A	WT		
128	M	SNAC	84		N/A	N/A	N/A	WT		
129	M	SNCC non keratinizing	75	nasal/paranasal	negative	negative	MMR-P	TP53	c.797G>A	p.G266E
								EGFR	c.2239_2240delinsCC	p.L747P
130	M	ISP	53	nasal	negative	N/A	MMR-P	EGFR	c.2311_2319dup	p.N771_H773dup
131	M	SNAC	43	paranasal	negative	N/A	MMR-P	MAP2K1	c.159T>G	p.F53L
132	M	SNSCC sarcomatoid	53	nasal	negative	negative	N/A	WT		
133	M	ISP	41	nasal/paranasal	negative	negative	MMR-P	EGFR	c.2306_2307insCTCCAGCGT	p.V769_D770insSSV
134	M	SNSCC keratinizing	66	paranasal	negative	negative	MMR-P	TP53	c.742C>T	p.R248W
135	M	SNSCC keratinizing	64	paranasal	negative	negative	MMR-P	WT		
136	F	SNSCC keratinizing	53	nasal	negative	negative	dMMR	EGFR	c.2303_2311dup	p.S768_D770dup
								TP53	c.527G>T	p.C176F
137	F	SNSCC keratinizing	70	nasal	positive	negative	dMMR	TP53	c.404G>C	p.C135S
								TP53	c.869G>A	p.R290H

All varients >5% with coverage > 200x , * AF < 5%
