# Supplementary material for: Mismatch Repair Deficiency and Somatic Mutations in Human Sinonasal Tumors
Source: Cancers (Basel). 2021 Dec 2;13(23):6081. doi: 10.3390/cancers13236081 (PMC8657279; doi:10.3390/cancers13236081)
Supplement: Supplementary file 1 [file cancers-13-06081-s001.zip › Table S4_Mutation frequency in sinonasal tumor cohort.pdf]

Table S4: Mutation frequency in sinonasal tumor cohort

	Subtype																									
	SNSCC keratinizing		SNSCC non-keratinizing		SNSCC associated with ISP		SNSCC sarcomatoid		SNSC verrucous		ISP		ESP		OSP		ACC		SNAC		ITAC		SNUC		SNEC	
	N	N %	N	N %	N	N %	N	N %	N	N %	N	N %	N	N %	N	N %	N	N %	N	N %	N	N %	N	N %	N	N %
**TP53**	21	67.7%	1	12.5%	8	80.0%	0	0.0%	0	0.0%	0	0.0%	1	16.7%	0	0.0%	0	0.0%	5	45.5%	2	33.3%	1	9.1%	3	33.3%
**EGFR**	2	6.5%	3	37.5%	7	70.0%	0	0.0%	0	0.0%	25	89.3%	0	0.0%	0	0.0%	0	0.0%	0	0.0%	0	0.0%	0	0.0%	0	0.0%
**PIK3CA**	0	0.0%	1	12.5%	2	20.0%	0	0.0%	1	100.0%	0	0.0%	0	0.0%	0	0.0%	1	9.1%	1	9.1%	0	0.0%	1	9.1%	1	11.1%
**KRAS**	1	3.2%	1	12.5%	2	20.0%	0	0.0%	0	0.0%	0	0.0%	0	0.0%	1	100.0%	0	0.0%	0	0.0%	1	16.7%	1	9.1%	0	0.0%
**CARD11**	0	0.0%	0	0.0%	0	0.0%	0	0.0%	0	0.0%	2	7.1%	0	0.0%	0	0.0%	0	0.0%	0	0.0%	0	0.0%	0	0.0%	0	0.0%
**NOTCH1**	3	9.7%	0	0.0%	0	0.0%	0	0.0%	0	0.0%	0	0.0%	0	0.0%	0	0.0%	0	0.0%	0	0.0%	0	0.0%	0	0.0%	0	0.0%
**CDKN2A**	0	0.0%	0	0.0%	1	10.0%	0	0.0%	0	0.0%	2	7.1%	0	0.0%	0	0.0%	0	0.0%	0	0.0%	2	33.3%	0	0.0%	0	0.0%
**NFE2L2**	1	3.2%	0	0.0%	2	20.0%	0	0.0%	0	0.0%	1	3.6%	0	0.0%	0	0.0%	0	0.0%	0	0.0%	0	0.0%	0	0.0%	0	0.0%
**PTEN**	2	6.5%	1	12.5%	1	10.0%	0	0.0%	0	0.0%	0	0.0%	0	0.0%	0	0.0%	0	0.0%	0	0.0%	0	0.0%	0	0.0%	0	0.0%
**BRAF**	0	0.0%	1	12.5%	0	0.0%	0	0.0%	0	0.0%	0	0.0%	0	0.0%	0	0.0%	0	0.0%	0	0.0%	0	0.0%	0	0.0%	1	11.1%
**MET**	0	0.0%	0	0.0%	1	10.0%	0	0.0%	0	0.0%	0	0.0%	0	0.0%	0	0.0%	0	0.0%	0	0.0%	1	16.7%	0	0.0%	0	0.0%
**FGFR3**	0	0.0%	0	0.0%	1	10.0%	0	0.0%	0	0.0%	0	0.0%	0	0.0%	0	0.0%	0	0.0%	0	0.0%	0	0.0%	0	0.0%	0	0.0%
**HRAS**	1	3.2%	0	0.0%	0	0.0%	0	0.0%	0	0.0%	0	0.0%	0	0.0%	0	0.0%	0	0.0%	0	0.0%	1	16.7%	0	0.0%	0	0.0%
**IDH2**	0	0.0%	0	0.0%	0	0.0%	0	0.0%	0	0.0%	0	0.0%	0	0.0%	0	0.0%	0	0.0%	0	0.0%	0	0.0%	1	9.1%	0	0.0%
**STAT3**	0	0.0%	0	0.0%	0	0.0%	0	0.0%	0	0.0%	1	3.6%	0	0.0%	0	0.0%	0	0.0%	0	0.0%	0	0.0%	0	0.0%	0	0.0%
**FGFR2**	0	0.0%	1	12.5%	0	0.0%	0	0.0%	0	0.0%	0	0.0%	0	0.0%	0	0.0%	0	0.0%	0	0.0%	0	0.0%	1	9.1%	0	0.0%
**C15orf23**	1	3.2%	0	0.0%	0	0.0%	0	0.0%	0	0.0%	0	0.0%	0	0.0%	0	0.0%	0	0.0%	0	0.0%	0	0.0%	0	0.0%	0	0.0%
**KEAP1**	1	3.2%	0	0.0%	0	0.0%	0	0.0%	0	0.0%	0	0.0%	0	0.0%	0	0.0%	0	0.0%	0	0.0%	0	0.0%	0	0.0%	0	0.0%
**MAP2K1**	0	0.0%	0	0.0%	0	0.0%	0	0.0%	0	0.0%	0	0.0%	0	0.0%	0	0.0%	0	0.0%	1	9.1%	0	0.0%	0	0.0%	0	0.0%

SNSCC = sinonasal squamous cell carcinoma, ISP = inverted sinonasal papilloma, ESP = exophytic sinonasal papilloma, OSP = oncocytic sinonasal papilloma, ACC = adenoid cystic carcinoma, SNAC = adenocarcinoma with no intestinal nor salivary gland differentiation, ITAC = intestinal type adenocarcinoma, SNEC = sinonasal neuroendocrine carcinoma, sinonasal undifferentiated carcinoma = SNUC
