# Supplementary figures and images for: Mismatch Repair Deficiency and Somatic Mutations in Human Sinonasal Tumors
Source: Cancers (Basel). 2021 Dec 2;13(23):6081. doi: 10.3390/cancers13236081 (PMC8657279; doi:10.3390/cancers13236081)

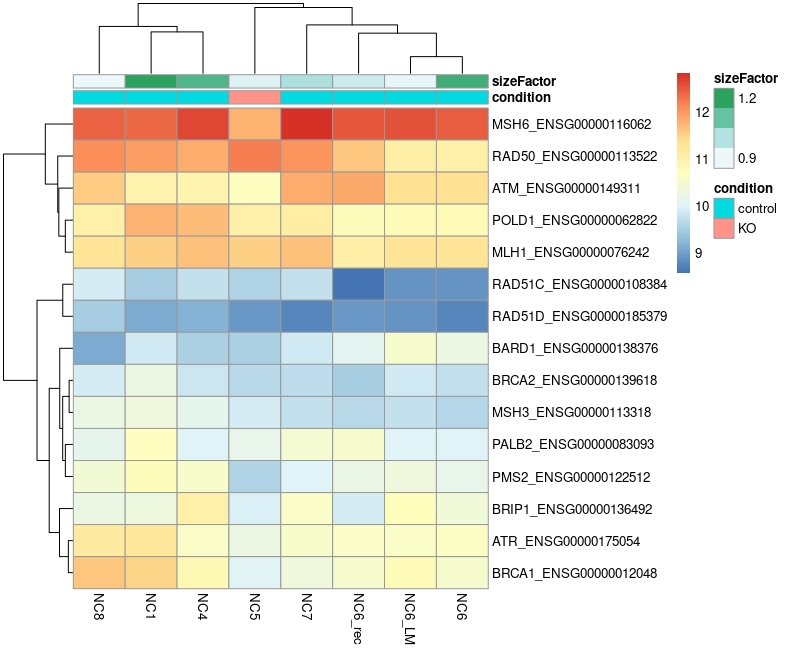

Supplement: Supplementary file 1 [file cancers-13-06081-s001.zip › Figure S1_mRNA expression of DNA mismatch repair and homologous repair (HR) genes.jpeg]
